# Case Report: Capitalizing on Development and Activity-Dependent Plasticity, an Interaction With Pediatric-Onset Spinal Cord Injury

**DOI:** 10.3389/fped.2022.804622

**Published:** 2022-03-29

**Authors:** MacKenzie Goode-Roberts, Kathryn Noonan, Danielle Stout, Margaret Calvery, Kyle Brothers, Nicole Williams Doonan, Andrea L. Behrman

**Affiliations:** ^1^UofL Health, Frazier Rehab Institute, Kosair Charities Center for Pediatric NeuroRecovery, Louisville, KY, United States; ^2^Norton Children’s Medical Group, Louisville, KY, United States; ^3^Department of Pediatrics, University of Louisville, Louisville, KY, United States; ^4^Norton Children’s Research Institute, Affiliated With the University of Louisville School of Medicine, Louisville, KY, United States; ^5^Department of Neurology, Gillette Children’s Specialty Healthcare, St. Paul, MN, United States; ^6^Department of Neurological Surgery, Kosair Charities Endowed Chair in Pediatric NeuroRecovery, University of Louisville, Louisville, KY, United States

**Keywords:** infancy, plasticity, spinal cord injury (SCI), activity-based restorative therapies, development

## Abstract

**Background:**

Spinal cord injury (SCI) in infancy halts typical development secondary to paralysis/paresis and the limited ability to engage with the environment. Traditional therapies further restrict a child via bracing, equipment, and medications. In contrast, activity-based restorative therapies (ABRT) promote activation of the neuromuscular system below the level of injury and affords a more typical sensorimotor experience.

**Case Description:**

A premature male infant exhibiting hypotonia, poor head control, and extremity weakness was diagnosed at age 5 months with a remote incomplete upper cervical SCI based on magnetic resonance imaging (MRI), presumed to have occurred perinatally. From 4 to 15 months of age, he received physical, occupational and speech therapies. Enrolled in an ABRT program at 15 months, he was unable to sit, pull-to-stand, stand, or walk and had upper extremity impairments. Results of the Bayley-III Scales of Infant and Toddler Development revealed gross and fine motor scores consistent with a 4-month-old.

**Methods:**

Activity-based restorative therapies was provided 5 day/week: 1.5 h of activity-based locomotor training and 1 h of activity-based occupational therapy.

**Results:**

Activity-based restorative therapies are reported for 177 sessions and are on-going. Improvements are noted in trunk control, standing, walking, grasp, in-hand manipulation, and associated kinematics. Bayley-III fine motor score improved to that of a 16-month-old and gross motor score to that of a 7-month-old.

**Discussion:**

While the two treatment periods (i.e., 4–15 months old and 15–24 months) were each ∼9 months, the child’s accelerated progress toward typical development during the latter, ABRT period is noteworthy. In comparison to the period of traditional therapies in which paralysis was compounded by a restrictive environment and compensation, ABRT provided a potentially rich sensorimotor experience with an emphasis on active weight-bearing and proper kinematics to activate the neuromuscular system below the lesion in an age-appropriate, task-specific context of activities. Improved physical capacity enabled exploration more typically associated with development at this age expanding the positive impact to other developmental domains.

## Introduction

Rapid musculoskeletal growth and development characterize the first year of human life. Spinal cord injury (SCI), whether neonatal or in infancy, halts typical development. Paresis and paralysis of trunk and limb muscles results in the inability to move, explore, and learn via interactions with the environment. Traditional physical rehabilitation in young children is typically 1 − 2x/week. With paralysis assumed to be permanent (1, 2), therapists apply prone positioning targeting head control, promote the developmental sequence, and compensate for trunk and limb paralysis by focusing on muscles above the injury recommending equipment (e.g., braces, stander) to achieve functional sitting, standing, and mobility. With spasticity resulting from upper motor neuron lesions, physicians may introduce botulinum toxin (Botox) as an anti-spasticity medication. With SCI and paralysis, the inability to move decreases and alters the sensorimotor experiences of a child essential to development. This is compounded when using braces, standers, and medications, further restricting mobility, and altering the sensorimotor experience.

Activity-based restorative therapies (ABRT), e.g., activity-based locomotor training (AB-LT) (3–6), neuromuscular electrical stimulation (7, 8), transcutaneous spinal stimulation (9, 10), in comparison, target activation of the neuromuscular system below the lesion. During ABRT, emphasis is on facilitating task-specific kinematics during repetitive training, 5 days/week. Neither anti-spasticity medications nor trunk or limb braces are used to afford apt sensory input during training in the clinic and home/community. During the delivery of ABRT with children, therapists use age-appropriate play as a driving and meaningful context for the child. Thus, play is an essential tool capitalizing on the child’s inherent motivation and intent to move necessary to engage them and achieve therapeutic goals (11–13). We report the case of an infant with a cervical SCI presumed to have occurred *in utero* or at the time of birth who received traditional therapies (8 days to 15 months of age), followed by an intensive and on-going course of ABRT (reporting on 15–24 months of age) with the perspective that “experience” guided by activity-dependent plasticity matters to advance and promote a more typical course of development.

Only one other case report has been published to date describing rehabilitation of an infant with an intra-uterine SCI. The child, however, was treated more acutely within the first year following injury with a combination of ABRT and compensation strategies (14). Pape (15) also provides a brief description of rehabilitation of an infant with a cervical SCI. The child’s injury, however, occurred during delivery and treatment began at 3 years old with a therapeutic regime focused on neural activation below the injury using neuromuscular electrical stimulation. With neonatal SCI being rare (15), and only one other case study describing rehabilitation beginning in the acute stage, this current study follows the case of a child with a neonatal SCI who received ABRT in the chronic phase. This case thus adds to the limited available literature to guide clinical decision making.

## Case Description

**FIGURE 1:**
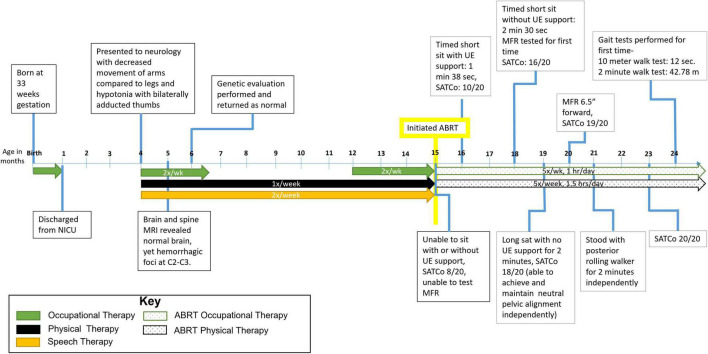
Timeline from birth to 24 months of age. Patient initiated activity-based restorative therapy (ABRT) at 15 months. Arrows indicate therapies the patient was receiving and number of times per week he participated. UE, upper extremity; SATCo, segmental assessment of trunk control; MFR, modified functional reach.

**FIGURE 2:**
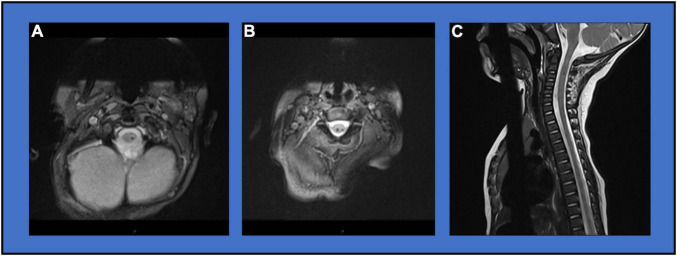
Diagnostic Imaging. **(A)** MRI Axial MEDIC image showing focal T2 hypointense signal at the C1 level. **(B)** MRI Axial MEDIC image showing focal T2 hypointense signal at the C2/3 level. **(C)** MRI Sagittal T2 image showing mixed hypo- and hyper-intensity lesion with volume loss at the C1/2 level.

A male child was born at 33 weeks gestation following a pregnancy complicated by premature spontaneous rupture of membranes at 29 weeks and bicornate uterus. Mother was hospitalized on bedrest until spontaneous onset of labor at 33 weeks gestation. Delivery was by non-instrumented vaginal delivery in the cephalic position. He was admitted to the neonatal intensive care unit (NICU) at birth for management of respiratory distress and prematurity. He required positive pressure support for 3 days. He received inpatient occupational therapy for treatment of congenital positional plagiocephaly, torticollis, and mild positional bilateral foot deformities (Figure 1). He was discharged home at 3 weeks feeding orally on demand. He presented to neurology clinic at 4 months old for evaluation of hypotonia and motor delays with persisting torticollis and plagiocephaly. Mild feeding difficulties were reported without signs or symptoms of aspiration. There were no respiratory symptoms. Initial neurological examination was notable for axial hypotonia, poor head control, proximal and distal weakness of all extremities affecting arms more than legs, bilateral thumb-in-palm posture, normal deep tendon reflexes, and upgoing toes. Normal diagnostic studies at age 5 months included brain MRI, chromosomal microarray with limited high-resolution chromosomes, serum creatine kinase, and thyroid studies. Initial spine MRI without contrast at age 5 months identified small foci of T2 hypointense signal at the C1 and C2/3 levels as well as possibly at the T11 level consistent with small hemorrhagic foci (Figures 2A,B). Intervening mild volume loss within the spinal cord at the C1/2 level associated mild T2 hyperintensity was also seen (Figure 2C). Repeat spine MRI with and without contrast at ages 6 months and 1 year showed stable findings. Postcontrast images demonstrated no abnormal enhancement. The possibility of a small vascular malformation was considered and dismissed based on follow up imaging. The presence of chronic microhemorrhage and cord atrophy were most consistent with a remote SCI. In the absence of a known traumatic mechanism, the injury was presumed to occur *in utero* or around the time of birth. The patient’s neurological examination evolved as expected over the first year of life with development of spasticity and hyperreflexia with clonus in extremities. Bowel and bladder function remained within normal limits for age.

From 4 to 15 months, he received therapies focused on head control in prone, developmental sequence, fine motor control, and oral motor control. Medical record review provides insight into interventions and positions the patient was placed in during usual occupational and physical therapy care. The developmental sequence was used for positioning during therapeutic activities, as well as an abdominal binder where trunk control was lacking. In summary, the patient was primarily treated in prone, quadruped, supine or facilitated sitting with an abdominal binder. There was no indication of the specific methodology used during intervention but, the focus at that time was reported as bed mobility, weightbearing on arms in a supported quadruped position, and sitting balance/posture with an abdominal binder. Physical therapy goals included: demonstration of midline posture of trunk and head with assist of abdominal binder and minimal assist by provider for 3 mins, demonstrate independent symmetrical load through upper extremities in prone with head upright for 10 s, demonstrate alternating weight shift and upper extremity reach in prone position with head right to midline with minimal assist, independent roll back to tummy with head righting. Occupational therapy goals included: sitting with minimal support while reaching outside of base of support to place items in open container, sit with moderate assist and activate cause and effect toy on 2/3 trials, will imitate play and clap/bang toys together in midline on 2/3 trials, will oppose thumb and fingers to pick up small crackers with minimal assist.

Medical management included recommendations for Botox injections to gastrocnemius muscles, ankle foot orthotics (AFOs), abdominal binder use, wrist splint use and a standing frame. The family consistently used the hand splints for the infant but use of the AFOs was inconsistent due to severely increased plantar flexion posturing. A standing frame was used in the home with trunk, pelvic, lower extremity (LE) and foot supports.

He presented to an outpatient, pediatric ABRT program for evaluation and treatment at 15 months old. His torticollis and feeding concerns had resolved. At initial evaluation, the child was unable to sit (with or without upper extremity support), grasp objects appropriately, complete full shoulder flexion, pull to stand, stand, or walk. His initial Segmental Assessment of Trunk Control (SATCo) (16, 17) score was 8/20, meaning that with external support at the inferior angle of the scapula and pelvis, he could maintain trunk alignment above the support in response to trunk perturbations at the sternum. He exhibited an excessive poster pelvic tilt and thoracolumbar kyphosis in short-sitting with support needed to prevent falling. Vertical pelvic alignment could be achieved with manual facilitation.

He demonstrated hyper reflexive patellar deep tendon reflexes and a positive Babinski, bilaterally. Clonus was observed, though specific testing did not elicit the response. With manual support, he could “weight-bear” through LEs exhibiting both excessive genu recurvatum and plantar flexion dominated by extensor tone. With support at the axillae, he initiated steps with plantar flexion posturing (no dorsiflexion) and LE scissoring (i.e., one LE crossing across over the other). Upper extremity (UE) posturing was predominately in wrist and finger flexion with ulnar deviation and avoidance of grasp of objects. Volitional finger extension through partial range was observed, however, the index finger remained relatively flexed bilaterally. He could not flex his shoulder above nipple line and would not weight-bear through an extended wrist. At 15 months of age, the Bayley-III Scales of Infant and Toddler Development (18) revealed gross and fine motor scores consistent with a 4-month-old.

## Methods

The child initiated and is enrolled in an outpatient ABRT program (3, 4, 6, 19–21). Therapy is provided 5 day/week: 1.5 h of activity-based locomotor training (ABLT), 1 h on the treadmill and 30 mins of overground and community integration, and 1 h of activity-based occupational therapy (ABOT). During ABLT (5), the treadmill portion consisted of manually-facilitated stepping and standing emphasizing locomotor-and task-specific kinematics with the patient partially unweighted in a body weight support (BWS) system and harness. Treadmill speed was at 1.0 mph or below while BWS was assessed daily and set to lowest amount of support while maintaining kinematics. Manual facilitation and a circumferential strap with the harness were used initially at low ribs to provide support. Age-appropriate play and rhythmic songs were integrated to maintain patient engagement to stepping task while encouraging active participation of stepping and arm swing. Standing activities focused on skill recovery or acquisition in an area most lagging or rate-limiting. Examples include reaching overhead encouraging trunk extension and shoulder extension with appropriate digit extension, squat-to-stands, single leg standing, marching. Treatment off the treadmill (overground), capitalized on the patient’s activated neuromuscular system during sitting, standing, age-appropriate transitions (i.e., squat-to-stands, sit-to-stands), and walking, encouraging self-initiated movements, and typical task kinematics. Caregiver education targeted integration of principles into the home/community where parental choices of positions and activities supported clinical gains for greater practice and repetition. Progression of recommendations was an evolutionary process and mirrored the patient’s habilitation.

Activity-based occupational therapy sessions immediately followed ABLT daily and further focused on appropriate kinematics specifically of trunk and UEs but with a holistic, full body approach during sitting, standing and transitional tasks. Therapeutic activities focused on facilitating wrist and digit kinematics during age-appropriate play, performing “pull-to-stands,” active use of arms/hands, employing varied objects to facilitate task-specific kinematics for pinch and age-appropriate grasps, and encouraged weight bearing through an extended wrist. Trunk control was challenged and progressed simultaneous to all UE focused tasks. Formal re-evaluations occurred approximately every 20 sessions in both PT and OT.

## Results

Findings are reported through session 177 and 25 months of age as therapy continues. The child rapidly adjusted to the therapy schedule and attended ABRT daily (5x/week) with 95% adherence/attendance rate to scheduled therapy sessions (2% absences, i.e., 4/177 due to illness and 3% absences, i.e., 5/177 due to weather, transportation and medical appointments). There were no adverse events during the period. An orthopedic evaluation was sought due to atypical forefoot positioning (R > L), to confirm development of hip sockets without dysplasia, and to provide a baseline of spine alignment.

### Trunk Control Progression

**FIGURE 3:**
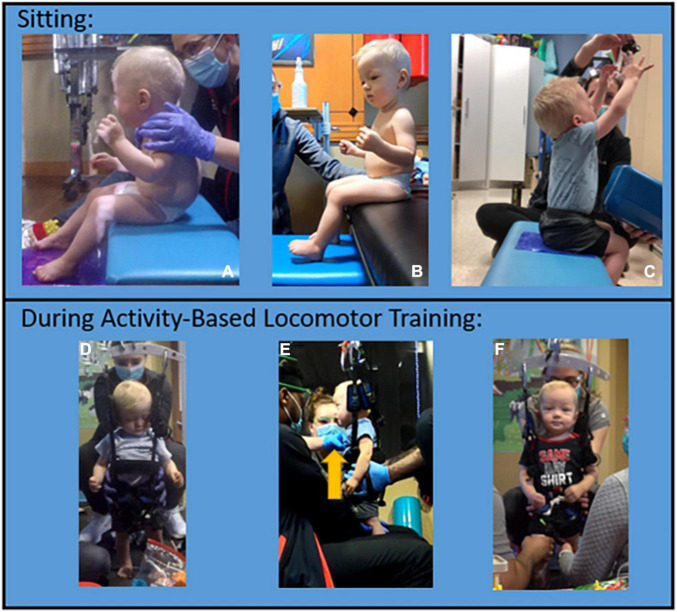
Trunk control progression. Sitting trunk control progression. **(A)** Initial evaluation: significant posterior pelvic tilt with kyphosis, unable to sit with or without upper extremity support. **(B)** Session 73: improved trunk and pelvic posture, the patient is able to maintain independently without upper extremity support. **(C)** Session 129: the patient can reach overhead with bilateral upper extremities to grasp object with maintenance of posture and balance. Trunk control progression during AB-LT: **(D,E)**. Initially, the harness strap was required at low ribs to assist with upright trunk posture and additional facilitation at the trunk was required for the patient to stay upright (see arrow in panel **E**). **(F)** Progression noted by removal of trunk strap during dynamic stepping activity with patient maintaining trunk alignment.

Trunk control improved to SATCo 20/20 (reactive control with no support), sitting independently without UE support for >2 mins, and modified functional reach (22) assessed reaching forward 6.5″, left/right 2.5″. The child now sits with a vertical pelvis, upright extended trunk, and can lift his arms overhead to hold a ball (Figures 3A–C). He can sit in modified ring/long sitting without UE support with posteriorly tilted pelvis and kyphotic trunk posture for 2 mins. While tailor sitting (legs flexed and crossed), he can achieve a vertical pelvis with fully upright trunk. Initially, the patient required harness strapping and overpressure at his trunk to achieve an upright posture in the treadmill environment. Indicative of a change in trunk control, the trunk strap was removed and he was able to maintain his upright posture (Figures 3D–F). In the first 20 sessions, the patient improved to prop-sit 30 s–1 min. At the 160-session evaluation, the patient could independently sit on a bench and play using bilateral UEs for an entire 60-min ABOT session.

### Upright Standing and Sit-to-Stand Progression

Timed stand with a pediatric posterior walker improved from unable to stand independently with device to >2 mins. In this position, he demonstrates genu recurvatum and variable foot alignment from foot flat to plantar flexion to supination/pronation. He can stand with his knees in neutral alignment to minimal flexion and feet flat on the ground, though inconsistently. During standing, he can voluntarily initiate knee flexion from a hyperextended position to a more neutral knee position. He can complete a pull-to-stand independently with inappropriate LE kinematics, specifically adduction and internal rotation of the right hip and genu recurvatum bilaterally. From an elevated bench, he can initiate sit-to-stand with weight shift forward over his feet requiring assistance at the LEs to transition fully to stance.

### Self-Mobility Progression

**FIGURE 4:**
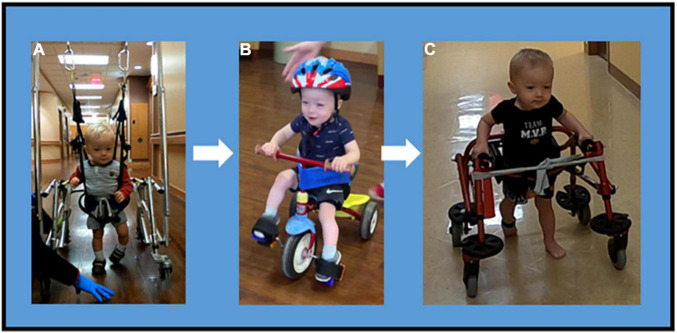
Mobility progression. Mobility Progression over time during ongoing course of activity-based restorative therapy. **(A)** Ambulation was initiated with use of a walker, harness and body weight support. The patient required assist for propulsion and continued to demonstrate a scissoring gait pattern with genu recurvatum and excessive plantarflexion. **(B)** At session 90, the patient can independently propel a tricycle with adaptations for pelvic alignment, for safety (seat belt) and foot strapping. **(C)** Patient progressed to independent reciprocal stepping in a reverse rolling walker with hip guides and sling seat. This is the primary means of mobility.

Prior to achieving walking, a modified tricycle was introduced, session 90, as a means of community mobility. The patient progressed from being unable to step with an assistive device and requiring trunk assist at axillae, to taking reciprocal steps while in a walker with harness and BWS with assist for walker propulsion, to independent ambulation using a reverse rolling walker with sling seat and pelvic guide for safety. He can navigate obstacles, turns and inclines. At session 177, gait speed was recorded at 0.8 m/s using the 10-m walk test (23) and gait endurance was 42.8 m during the 2-min walk test (24). He continues to demonstrate genu recurvatum, however, with improved foot placement (decreased scissoring) and increased step length (Figure 4).

### Home Recommendation Progression

The child’s stander was modified to challenge trunk control, decreasing the height of trunk support initially and over time completely removing the trunk strap and having only pelvic, LE and foot straps. A bench was recommended so the patient could practice short sitting during play and snacks. Notably, a tricycle was introduced as a means of independent mobility emphasizing reciprocal LE engagement. Once appropriate, a recommendation was made for walking in a walker with overhead suspension. This progressed to independent use of a posterior rolling walker with pelvic assist.

### Upper Limb and Hand Manipulation Progression

**FIGURE 5:**
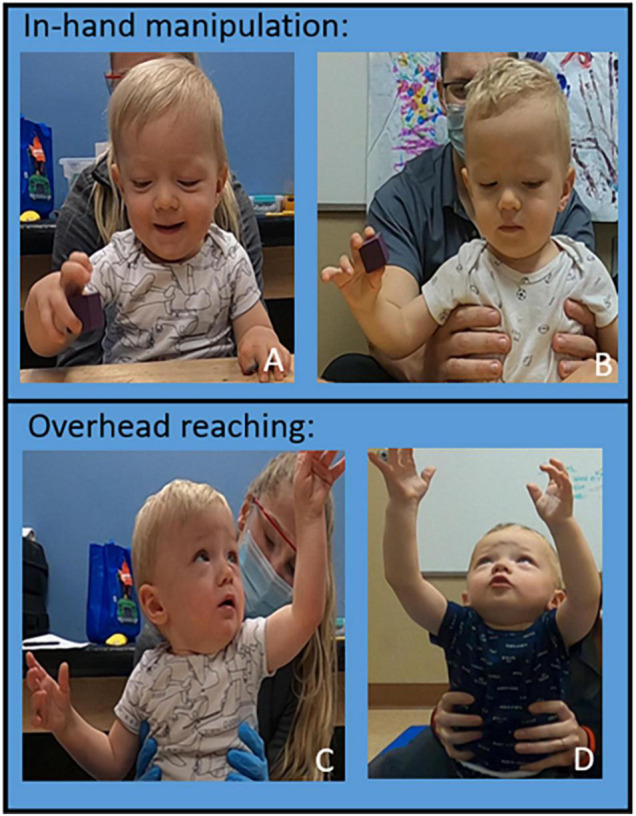
Upper Extremity Progression. In hand manipulation progression. **(A)** Session 40: inappropriate kinematics of digits two and three performing in-hand manipulation. **(B)** Session 140: appropriate kinematics of digits two and three performing in-hand manipulation independently. Overhead reaching progression: **(C)** Session 40. The patient can fully flex his elbow, pronate the forearm without shoulder abduction, and able to flex his shoulder greater than 90° with appropriate kinematics and trunk support at inferior scapula. **(D)** Session 160 the patient flexes his shoulder fully to reach overhead with appropriate kinematics and decreased trunk support (low ribs).

The patient improved in-hand manipulation from grasping a 1″ block with inappropriate kinematics to grasping the block with appropriate kinematics, specifically of first- and second-digit extension (Figures 5A,B). The patient improved overhead reach in bilateral UEs: able to grasp a half pound weight, fully flex elbow, pronate forearm, flex shoulder overhead with appropriate kinematics except for wrist and fingers in his right UE and complete movement fully with appropriate kinematics on the left (Figures 5C,D). Weight bearing has improved to palmar weight bearing with independent elbow, wrist, and digit extension. At initial evaluation, the patient’s mother shared concerns if her son would be able to feed himself as he was only able to “finger-feed” with inappropriate grasp and lack of coordination. He now consistently utilizes a mature pincer and pad-to-pad grasp for self-feeding and has fed himself an entire meal with a fork. He can independently hold a cup with a straw with sufficient strength to maintain grasp when the cup is full. Initially, the patient reacted to different sensations with tactile defensiveness. Hypersensitivity and avoidance were present throughout messy play experiences such as playdough, finger paint, water play, and food play. The patient now engages and enjoys play throughout sessions utilizing bilateral UEs to engage with various textures throughout tactile play. At the 160th session, the patient demonstrated the ability to perform the following grasps: pad to pad, mature pincer, gross, cylindrical, digital pronated for a writing instrument, and isolated 2nd digit extension. The patient engages in bilateral UE play reaching forward, lateral, and overhead inside and outside base of support with supervision for safety during reaching at the limits of stability. The patient can extend digits to grasp small, medium, and large sized objects with intermittent assist (less than 25% of the time) to correct 2nd digit kinematics for full extension. The patient follows verbal cues to address 2nd digit extension approximately 50% of the time.

### Play Assessment

We report on changes in self-selected play at the 20th and 160th session evaluation, adapted from the PACMI (25). Observation of self-initiated play at 20 sessions revealed the patient restricted to two positions for play: (1) manually facilitated long-sitting with trunk kyphosis and bilateral UE support and (2) prone on forearms. At the 160th session, the patient exhibited a wider range of positions for play and freely transitioned from sitting to prone on forearms to side lying. Improved second finger digit extension was noted at the 160th session, with fewer times using inappropriate flexion.

### Musculoskeletal Assessment

At 17 months, X-rays revealed no scoliosis, hips in socket, and normal range at hips, knees, and ankles with metatarsus primus varus of the left foot. Orthopedic follow-up at 23 months showed no change except improved forefoot positioning.

### Developmental Assessment

Bayley Scales of Infant and Toddler Development (Bayley-lll) (18) was utilized to assess developmental changes across ABRT. Communication (receptive and expressive) and Motor (fine and gross) domains were measured with conversion to both standard score and developmental age. Standard scores across gross motor abilities remained stable between time point 1 (initial evaluation) and 2 (∼session 150). While fine motor abilities significantly improved [SS (standard score) = 55 at time point 1, SS = 75 at time point 2], all standard scores remained well below expected levels. Analysis at the standard score level does not capture the significant changes in development noted in therapy, at home, and on assessment. Across the 9 months of intervention, gross motor abilities improved by 11 months, 10 days. Instead of using the term “going to therapy,” the patient spontaneously now reports, “going to go walking” and “I walk” referring to treadmill stepping during AB-LT sessions. Fine motor abilities improved almost 3 months, nearly doubling his developmental gains in this domain across 9 months of intervention in comparison to only 4 months, 10 days of development across his first 15 months of life. His mother noted on interview, “he insists on getting things for himself now.”

Of the many developmental changes noted in the patient, the organic spilling of developmental gains across domains was increasingly noted. With his newfound motor skills, the patient demonstrated rapid increases in social and emotional communication. Initially described by his parent as a “reserved” child who “needed to get comfortable with new people”, he now would “walk down the hall” initiating interaction with unknown others, saying “hello and bye”, turning his head and maintaining gaze until eye contact was reciprocated.

Play and playfulness were reported for the “first time” in the child’s life. After 3 months of ABRT his mom reported, “His personality I feel like is developing a little bit more now too, just based on the fact that he can go get the things that he wants, and he can play with my daughter a little bit more. Like they can play together while I’m you know, making dinner or something and she can interact or engage with him a little bit more, because he can move.”

## Discussion

This is the only known and detailed case report that demonstrates improvements in trunk control, ambulation and upper extremity capacity in a child with a SCI occurring *in utero* or at birth with standardized ABRT begun after 1 year since injury (i.e., a chronic stage post-SCI). At 1-year post-SCI, there is little expectation of improvements with interventions due to the chronicity of injury. In addition, in the absence of a known traumatic mechanism, the injury in this case was presumed to occur *in utero* or around the time of birth. Neonatal spinal cord injuries are most often associated with difficult or traumatic deliveries and are rarely reported following uncomplicated delivery in the cephalic position (26, 27). Complicated deliveries that result in SCI are also at a high risk for death and if survival occurs, it is with significant physical impairments and paralysis (27). Furthermore, this infant was not diagnosed with a SCI until 5 months of age. The parents initiated further medical examination due to their perception that development was not normal and “something was not right.” An MRI was critical to the diagnosis. This case highlights the unique context of rehabilitation with (1) development interrupted and delayed due to neonatal, cervical SCI with delivery in the cephalic position, (2) on-going maturation, and (3) delivery of neuro-therapeutic interventions beginning 1-year post-SCI. The child’s subsequent gains in neuromuscular capacity afforded new interactions with his environment and thus has propelled development forward across multiple domains.

Initial standardized assessments at 15 months of age revealed impairments associated with the patient’s injury restricted his gross and fine motor development. The Bayley-III, clinical objective findings, and parent report, reveal acceleration of developmental gains across domains during the period of ABRT. These gains included physical goals related to the intervention as well as a broader range of impact within the developmental spectrum. In comparison to traditional rehabilitation in which medication and compensation are the therapeutic strategies to meet immediate functional goals, ABRT provided rich sensorimotor experiences during repetitive and progressive practice based on the concepts of activity-dependent plasticity and the role of the spinal cord in controlling movements (20, 28). In this context, “activity drives plasticity and plasticity (circuitry modification) drives the behavior” (21). Acknowledging that development and maturation are not linear, ABRT converged with the typical maturation experience and enabled this patient with delayed motor development to physically explore his world via ambulation, tricycle pedaling, sitting upright independence, and fine motor manipulation providing a rich context for development (12, 13). Consistent with development, the repetitive practice in ABRT mimics the extensive number of repetitions children use as they develop new motor skills and learn how to adapt to their environment (29).

Ordinarily, a child would demonstrate a 9-month change in developmental abilities across the initial 4–15 months of life and another 9-month change in abilities during the following 15–24 month time period. When this patient arrived for initial evaluation at 15 months of age, he was unable to complete anticipated developmental milestones such as sitting independently (typically develops between 5 and 8 months of age), cruising (typically develops between 7 and 12 months), standing (typically develops between 9 and 14 months), or walking (typically develops between 11 and 15 months) and already experiencing significant developmental delay. The maturation that took place and the therapies he was receiving during that initial 9-month period did not result in a positive change toward typical development whereby he was able to perform age-appropriate motor tasks. ABRT may facilitate a resolution of neuromuscular incapacity that occurs secondary to SCI as the timing of an injury and initiation of ABRT may have a strong impact on the developmental landscape (30). Further, the improved neuromuscular capacity positively interacted with developmental delay and maturation. Maturation may certainly be a contributing component to change and improvement, but we suggest that maturation requires guidance by experience-dependent therapies. SCI, paralysis, and the associated impairments and inabilities cannot be resolved solely by maturation. For a child with a neonatal-onset SCI, experience and the type of experience likely matters to positively advance habilitation in the context of and interaction with development delayed by injury, physical limitations, and on-going maturation. The relevance of sensorimotor experience and deprivation for development has been detailed in the scientific literature of neglect highlighting the impact of neuromuscular disorders. Disorders of the neuromuscular system resulting in immobility and limiting exploration impede cognitive development (12, 13, 31, 32). Within this setting, ABRT may more effectively align to promote the typical developmental experience (e.g., facilitated, active standing and stepping on a treadmill vs. passive standing in a stander) of a child with SCI, thus, enabling development across multiple domains (12, 30).

The patient’s gains in motor capacities and control reverberated across developmental domains with self-initiated “classic” and complex skill demonstrations observed and reported by the family across language, social/emotional, adaptive, and play domains. The patient’s gains across developmental domains were observed across environments including therapy, home, and within the community. Finally, developmental gains consistent with developmental theory were not coached, practiced, or suggested, rather they were the natural outcroppings of the increased capacity and ability that had been created via the ABRT intervention and its delivery in an age-appropriate context of play. The patient’s awareness of his new-found abilities became apparent where he would state, “I do” or “me do.” He would become increasingly frustrated when therapists or his parents would attempt to assist him with an activity that he deemed himself independent.

While group comparisons and the randomized clinical trial (RCT) are viewed as the gold standard for scientific evidence, the rarity of cervical SCI whether neonatal or presented with cephalic delivery challenges the possibility of conducting RCTs in this population. Group settings and comparisons are highly unlikely (15) as the incidence of children with SCI either *in utero* or during cephalic births is extremely rare (27) relative to the already low percentage of pediatric-onset SCI (3–5% injured when <15 years of age) occurring within the approximate 10–11,000 injured individuals per year (33). Lesion heterogeneity, presentation variability, and multiple geographical sites of birth makes group comparisons impossible and creates significant risks to the validity of group research. A direct comparison between therapeutic approaches via group comparisons is not feasible. A comparison of sequential findings relative to the timing and introduction of different therapeutic approaches is complicated by developmental delay, as well as ongoing developmental maturation, yet similar to single-subject design with interventions separated by wash-out periods. The rate of change in this child’s neuromuscular capacity relative to the shift in therapeutic approaches and the onset and intensity of ABRT provides at minimum face validity to its positive effect for this individual. Case reports or single subject design studies are thus viewed as appropriate for providing evidence to guide clinical-decision making (15) especially when standardized and well-described therapies (3, 5, 20, 34), and psychometrically-proven pediatric outcome measures (17, 18, 22, 24, 35) add to the validity of the intervention, clinical data and potential for future implementation.

While significant gains have been made by this child across the 9-month period of ABRT, therapeutic goals remain to reduce UE support during walking (i.e., progression or even elimination of an assistive device). While functional mobility remains a goal, how this child walks and his neuromuscular capacity to control his body without assistance of a walker or braces using more typical kinematics is paramount to expanding his options for continued exploration for participation as he ages. On-going ABRT will target such gains, as well as refined and consistent patterns of UE movement for successful manipulation with self-feeding, use of utensils, and manipulation of objects for play or writing. Physical and occupational therapists continue to re-evaluate progress, adjusting therapeutic goals to maximize the child’s neuromuscular capacity and developmental potential via ABRT. For infants injured due to a SCI and paralysis, early delivery of intensive ABRT may be a beneficial intervention to change and accelerate the trajectory of outcomes across domains (e.g., cognitive, motor, perception) during this pivotal period of growth and development.

## Data Availability Statement

The original contributions presented in the study are included in the article/supplementary material, further inquiries can be directed to the corresponding author.

## Ethics Statement

The studies involving human participants were reviewed and approved by University of Louisville Institutional Review Board. Written informed consent to participate in this study was provided by the participants’ legal guardian/next of kin. Written informed consent was obtained from the minor(s)’ legal guardian/next of kin for the publication of any potentially identifiable images or data included in this article.

## Author Contributions

MG-R oversaw the physical therapy clinical data collection and drafted the manuscript. KN and DS oversaw the occupational therapy clinical data collects and provided the manuscript edits. MC oversaw developmental psychology care and provided related input for this manuscript. KB provided input related to qualitative interviews and editing of the final draft. NWD oversaw neurology care prior to ABRT, provided medical diagnostic imaging, and provided the manuscript edits. AB oversaw all clinical and research interventions for this child and drafted and edited the manuscript. All authors contributed to the article and approved the submitted version.

## Conflict of Interest

AB was volunteer president of NeuroRecovery Learning, Inc., a non-profit organization that works to expedite the translation of new scientific findings for the care of those living with spinal cord injury. She was a co-author of Locomotor Training Principles and Practice (2011), receiving royalties from Oxford University Press. The University of Louisville licenses a specialized pediatric treadmill and body-weight support system and harness co-developed by AB. The remaining authors declare that the research was conducted in the absence of any commercial or financial relationships that could be construed as a potential conflict of interest.

## Publisher’s Note

All claims expressed in this article are solely those of the authors and do not necessarily represent those of their affiliated organizations, or those of the publisher, the editors and the reviewers. Any product that may be evaluated in this article, or claim that may be made by its manufacturer, is not guaranteed or endorsed by the publisher.

## References

[1] Chafetz RS Gaughan JP Calhoun C Schottler J Vogel LC Betz R Relationship between neurological injury and patterns of upright mobility in children with spinal cord injury. *Top Spinal Cord Inj Rehabil.* (2013) 19:31–41. 10.1310/sci1901-31 23678283 PMC3584792

[2] Calhoun CL Harvey LA. Mobility for children with spinal cord injury. In: Vogel LC Zebracki K Betz RR Mulcahey MJ editors. *Spinal Cord Injury in the Child and Young Adult.* London: Mac Keith Press (2014). p. 307–28.

[3] Behrman AL Nair PM Bowden MG Dauser RC Herget BR Martin JB Locomotor training restores walking in a nonambulatory child with chronic, severe, incomplete cervical spinal cord injury. *Phys Ther.* (2008) 88:580–90. 10.2522/ptj.20070315 18326054 PMC2390720

[4] Sadowsky CL McDonald JW. Activity-based restorative therapies: concepts and applications in spinal cord injury-related neurorehabilitation. *Dev Disabil Res Rev.* (2009) 15:112–6. 10.1002/ddrr.61 19489091

[5] Harkema SJ Behrman AL Barbeau H. *Locomotor Training: Principles and Practice.* New York, NY: Oxford University Press, Inc (2011).

[6] Dolbow DR Gorgey AS Recio AC Stiens SA Curry AC Sadowsky CL Activity-based restorative therapies after spinal cord injury: inter-institutional conceptions and perceptions. *Aging Dis.* (2015) 6:254–61. 10.14336/AD.2014.1105 26236547 PMC4509474

[7] Collins DF. Central contributions to contractions evoked by tetanic neuromuscular electrical stimulation. *Exerc Sport Sci Rev.* (2007) 35:102–9. 10.1097/jes.0b013e3180a0321b 17620928

[8] Dean JC Yates LM Collins DF. Turning on the central contribution to contractions evoked by neuromuscular electrical stimulation. *J Appl Physiol.* (2007) 103:170–6. 10.1152/japplphysiol.01361.2006 17463296

[9] Rath M Vette AH Ramasubramaniam S Li K Burdick J Edgerton VR Trunk stability enabled by noninvasive spinal electrical stimulation after spinal cord injury. *J Neurotrauma.* (2018) 35:2540–53. 10.1089/neu.2017.5584 29786465 PMC6205803

[10] Sayenko DG Rath M Ferguson AR Burdick JW Havton LA Edgerton VR Self-assisted standing enabled by non-invasive spinal stimulation after spinal cord injury. *J Neurotrauma.* (2019) 36:1435–50. 10.1089/neu.2018.5956 30362876 PMC6482915

[11] Piaget J Inhelder BR Weaver H Inhelder BR. *The Psychology of the Child.* New York, NY: Basic Books (1969).

[12] Lobo MA Harbourne RT Dusing SC McCoy SW. Grounding early intervention: physical therapy cannot just be about motor skills anymore. *Phys Ther.* (2013) 93:94–103. 10.2522/ptj.20120158 23001524 PMC3538987

[13] Harbourne RT Dusing SC Lobo MA McCoy SW Koziol NA Hsu LY START-play physical therapy intervention impacts motor and cognitive outcomes in infants with neuromotor disorders: a multisite randomized clinical trial. *Phys Ther.* (2021) 101:zaa232. 10.1093/ptj/pzaa232 33382406 PMC7910024

[14] Felter CE Neuland EE Iuculano SC Dean J. Interdisciplinary, intensive, activity-based treatment for intrauterine spinal cord infarct: a case report. *Top Spinal Cord Inj Rehabil.* (2019) 25:97–103. 10.1310/sci18-00025 30774293 PMC6368103

[15] Pape KE. Developmental and maladaptive plasticity in neonatal SCI. *Clin Neurol Neurosurg.* (2012) 114:475–82. 10.1016/j.clineuro.2012.01.002 22306423

[16] Butler PB Saavedra S Sofranac M Jarvis SE Woollacott MH. Refinement, reliability, and validity of the segmental assessment of trunk control. *Pediatr Phys Ther.* (2010) 22:246–57. 10.1097/PEP.0b013e3181e69490 20699770 PMC2927393

[17] Argetsinger LC Trimble SA Roberts MT Thompson JE Ugiliweneza B Behrman AL. Sensitivity to change and responsiveness of the segmental assessment of trunk control (SATCo) in children with spinal cord injury. *Dev Neurorehabil.* (2019) 22:260–71. 10.1080/17518423.2018.1475429 29787329

[18] Bayley N. *Bayley Scales of Infant and Toddler Develpment – Third Edition (Bayley-III) Administration Manual.* Bloomington, MN: Pearson (2006).

[19] Behrman AL Bowden MG Nair PM. Neuroplasticity after spinal cord injury and training: an emerging paradigm shift in rehabilitation and walking recovery. *Phys Ther.* (2006) 86:1406–25. 10.2522/ptj.20050212 17012645

[20] Roy RR Harkema SJ Edgerton VR. Basic concepts of activity-based interventions for improved recovery of motor function after spinal cord injury. *Arch Phys Med Rehabil.* (2012) 93:1487–97. 10.1016/j.apmr.2012.04.034 22920448

[21] Howland DR Trimble SA Behrman AL. Neurologic recovery and restorative rehabilitation. In: Vogel LC Zebracki K Betz RR Mulcahey MJ editors. *Spinal Cord Injury in the Child and Young Adult.* London: Mac Keith Press (2014). p. 399–410.

[22] Lynch SM Leahy P Barker SP. Reliability of measurements obtained with a modified functional reach test in subjects with spinal cord injury. *Phys Ther.* (1998) 78:128–33. 10.1093/ptj/78.2.128 9474105

[23] Pirpiris M Wilkinson AJ Rodda J Nguyen TC Baker RJ Nattrass GR Walking speed in children and young adults with neuromuscular disease: comparison between two assessment methods. *J Pediatr Orthop.* (2003) 23:302–7. 12724591

[24] Rossier P Wade DT. Validity and reliability comparison of 4 mobility measures in patients presenting with neurologic impairment. *Arch Phys Med Rehabil.* (2001) 82:9–13. 10.1053/apmr.2001.9396 11239279

[25] Kelly-Vance L Ryalls BO. A systematic, reliable approach to play assessment in preschoolers. *Sch Psychol Int.* (2005) 26:398–412.

[26] Goetz E. Neonatal spinal cord injury after an uncomplicated vaginal delivery. *Pediatr Neurol.* (2010) 42:69–71. 10.1016/j.pediatrneurol.2009.08.006 20004868

[27] Lee CC Chou IJ Chang YJ Chiang MC. Unusual presentations of birth related cervical spinal cord injury. *Front Pediatr.* (2020) 8:514. 10.3389/fped.2020.00514 33117760 PMC7550748

[28] Edgerton VR Courtine G Gerasimenko YP Lavrov I Ichiyama RM Fong AJ Training locomotor networks. *Brain Res Rev.* (2008) 57:241–54. 10.1016/j.brainresrev.2007.09.002 18022244 PMC2288528

[29] Adolph KE Cole WG Komati M Garciaguirre JS Badaly D Lingeman JM How do you learn to walk? Thousands of steps and dozens of falls per day. *Psychol Sci.* (2012) 23:1387–94. 10.1177/0956797612446346 23085640 PMC3591461

[30] Adolph KE Hoch JE. Motor development: embodied, embedded, enculturated, and enabling. *Annu Rev Psychol.* (2019) 70:141–64. 10.1146/annurev-psych-010418-102836 30256718 PMC6320716

[31] Johnson CP Blasco PA. Infant growth and development. *Pediatr Rev.* (1997) 18:224–42. 10.1542/pir.18-7-224 9203831

[32] Perry BD Pollard R. Altered brain development following global neglect in early childhood. In: *Proceedings of the Society for Neuroscience Annual Meeting.* New Orleans, LA (1997). 10.1017/s095457940300049x

[33] National Spinal Cord Injury Statistical Center. *2020 Annual Statistical Report - Complete Public Version.* Birmingham: National Spinal Cord Injury Statistical Center (2020).

[34] Harkema SJ Hillyer J Schmidt-Read M Ardolino E Sisto SA Behrman AL. Locomotor training: as a treatment of spinal cord injury and in the progression of neurologic rehabilitation. *Arch Phys Med Rehabil.* (2012) 93:1588–97. 10.1016/j.apmr.2012.04.032 22920456

[35] Ardolino EM Mulcahey MJ Trimble S Argetsinger L Bienkowski M Mullen C Development and initial validation of the pediatric neuromuscular recovery scale. *Pediatr Phys Ther.* (2016) 28:416–26. 10.1097/PEP.0000000000000285 27428576

